# Advancing Research Through Early-Career Scientists’ Publications and Training the Next Generation of Medical Editors: The First 10-Years of the International Journal of Medical Students

**DOI:** 10.5195/ijms.2022.1934

**Published:** 2022

**Authors:** Sebastian Diebel, Diego Carrion-Alvarez, Wah Praise Senyuy, Marina Shatskikh, Juan C. Puyana, Francisco J. Bonilla-Escobar

**Affiliations:** 1 Department of Family Medicine, Northern Ontario School of Medicine, Timmins, Ontario Canada.; 2 Departamento de Medicina Interna, ISSSTE Regional Monterrey, Monterrey, Nuevo León.; 3 Department of Medicine, Faculty of Health Sciences, University of Buea, Cameroon.; 4 Royal College of Surgeons in Ireland, Dublin, Ireland.; 5 School of Medicine, Department of Surgery, Professor of Surgery, Critical Care Medicine, and Clinical Translational Science, Director for Global Health-Surgery, University of Pittsburgh, Pittsburgh, PA, United States. Editorial Board Member, IJMS.; 6 Department of Ophthalmology; Institute for Clinical Research Education (ICRE), University of Pittsburgh, Pittsburgh, PA, United States. CEO, Fundación Somos Ciencia al Servicio de la Comunidad, Fundación SCISCO/Science to Serve the Community Foundation, SCISCO Foundation, Cali, Colombia. Grupo de investigación en Visión y Salud Ocular, VISOC, Universidad del Valle, Cali, Colombia. Editor in Chief, IJMS.

## Introduction

The International Journal of Medical Students (IJMS) has reached a new milestone. This historic issue will mark the final publication for the IJMS in the first 10-years of uninterrupted publications.^1^ The IJMS started following a discussion in 2009 at an international medical student congress where a conversation pertaining to student research was held. The discussion centered around the need for medical students to be acknowledged for their research, which in turn would lead to an improved research impact for the next generation in the medical-scientific community.^2^ By 2013 the ideas from the discussion at the congress had reached fruition and the IJMS published its first issue.

From its humble origins that started as an international project in Latin-America, the journal that is now an institution boasts a team of over 300 medical students, with representation from thirty-four different nationalities from all seven continents.^1,3,4^ This teamwork is being seen around the globe. In 2022, more than 250,000 website views were counted from more than 85,000 users (https://ijms.info/IJMS/statistics). In addition to the journal being widely disseminated, another accomplishment is that the journal has experienced long-term success when it comes to publishing. The IJMS is the longest-standing, non-interrupted international medical student journal that went from publishing three issues annually to four in 2021.^1^

Other accomplishments that are noteworthy in the journal’s first decade of existence include the indexing of multiple publications in PubMed Central (PMC).^1,5–11^ Another major accomplishment can be appreciated when looking at the journal’s success as it pertains to funding of the research that is published. Medical students under the mentorship of investigators with funding from the National Institute of Health (NIH) of the United States have specifically chosen the IJMS to publish their work.^12^

This year the IJMS also ventured into another side of the academic world with the first World Conference of Medical Students Research (WCMSR), which took place in November 2022 and aimed to reduce barriers such as time, transportation, registration costs, and limitations related to the role of medical students in this type of event. Held virtually, it attracted more than one hundred abstract submissions, which ended up in forty-one student presentations and over 1,200 viewers during that day, a number that is still growing, marking another staple in this ten-year celebration (First IJMS WCMSR link: https://www.youtube.com/watch?v=0JIMP5Fyl7s&t=0s). The editorial of the Conference has a better insight on the work behind this event and will be found in the Supplement of this volume, together with the abstracts from the Conference itself.^13^ The Supplement includes six different medical student-led conferences that are locally incentivizing the relevance of training in research and the visibility of student work, including:

- Abstracts of the 2022 American Physician Scientist Association (APSA) Northeast Regional Conference (NERC).^14^
- Abstracts of the Lagos State University Medical Students Association Research Conference.^15^
- Abstracts of the Medical University Congress of Mogi das Cruzes, COMUMC.^16^
- Abstracts of the Medical Academical Conference of Piauí (COMAPI) 2021.^17^
- Abstracts of the 2021 Yorkshire International Imaging and Interventional Radiology Symposium at the University of Leeds.^18^
- Abstracts of the Medical Academical Conference of Piauí (COMAPI) 2022.^19^

As the IJMS continues to progress and evolve, it is always seeking contemporary ideas in order to advance the Journal. As a result, 2022 saw the launch of MedEd Research Webinars. These are monthly research webinars that serve to develop a positive culture of virtual mentorship for medical students that may not have the opportunity to attend such events in their home countries. These webinars are specifically designed for medical students who want to improve their research skills. The overall mission and vision of the webinars is to be the leading diffusion platform for early career medical research (Link to the webinars: https://www.youtube.com/channel/UCoUYNIPrknOofTbP2WnioIA). These certainly are exciting times and the IJMS is additionally planning an online series that will be released in 2023 and disseminated on its YouTube platform entitled “Research Pathways.” It will be a space for top-tier researchers to share their stories and provide guidance on how to get there, together with their research career development.

This issue is truly a highlight of what the last 10-years has been for the IJMS. The Journal is publishing works from ten different countries that consists of seven original articles, two reviews, two case reports, one letter to the Editor, two editorials, one interview and four experiences.

The second editorial is a particularly important piece as it is a real call for global climate change efforts. This issue will highlight the third call for global action and collaboration for climate change. The IJMS remains the only medical student journal involved in this initiative. The editorial touches on the 2022 Report of the Intergovernmental Panel on Climate Change (IPPC) and the burden of the effects of climate change specially in Africa and other vulnerable regions.^20^

Some of the original articles in this volume continue to demonstrate both the tradition of international work and social accountability that has been on display in the IJMS in the past.^21^ The first original article to continue with the international theme was a study conducted by Bandyopadhyay et al., which set out to examine personality traits of medical students and how they evolve over the course of undergraduate medical training in India. Interestingly, the authors reported that the students became more introverted as time went on.^22^ Other international studies that examined low- and middle-income areas include the work by Johnson et al., which examined traumatic brain injuries in Honduras to better understand their etiology and severity, and finally the work by Amo-Tachie et al., which examined the health practices of blood donors in Ghana.^23,24^

As we head into 2023, it is important to note that the global pandemic of COVID-19 is still ever present. Two of the original studies had COVID-19-related research questions. Hardoon et al. look into the effects of COVID-19 lockdowns and fatality rates in the United-States during the first year of the global pandemic. They found that implementing safety protocols and lifting these protocols in phases reduced the spread of the virus, and ultimately the fatality rate.^25^ The second study, by Skoczek et al., examined the impact of the pandemic on medical learners and how it affected their education. The study found that International medical students reported more restrictions in their country and a larger mental health impact, while United States medical students were more likely to express a decrease in academic opportunities and academic performance.^26^

The last two original articles in this volume demonstrated novel techniques to answer fascinating research questions. El-Jack et al. performed research using the social media platform Reddit to gain better insight into the type of questions that patients may be posing about anesthesia.^27^ The last original article was conducted by Simões et al. and set out to explore if renal disease in the context of diabetic patients could be predicted by looking at specific gene expression.^28^

This issue also features two reviews. Rossi et al. performed a narrative review addressing the need for young physicians to be part of global surgery, a neglected but essential part of global health. They conclude with a strong proposal based on their setting and the need to have a broader approach to medical school curriculum.^29^ Campbell et al. performed a narrative review on the management for myelomeningocele, a study that explores and describes how the advancement in biomedical engineering can change these patients lives.^30^

Clinical cases in this issue were focused on anatomic findings. One article reported two cases of headaches associated with coughing, whose etiology was jugular venous insufficiency.^31^ The other case examined a post-mortem finding during an anatomy class, describing an aberrant right subclavian artery which occurs in less than 1% of the population.^32^

Understanding the importance of physicians’ role in addressing healthcare disparities, Lahiri et al. engaged with at-risk K-5th graders via an after school science program to increase students’ exposure to Science, Technology, Engineering, and Mathematics (STEM). Through hands-on projects the kids were inspired while participants gained skills to advocate for vulnerable patient populations in the future.^33^ Across Ghana, Dr. Amo-Tachie was struck by the disproportionate impact of a recent poliomyelitis outbreak. His insights into the significance of the outbreak, particularly in the context of concurrent stressors on the healthcare system posed by COVID-19, highlight the importance of remaining vigilant about controlling preventable infectious disease outbreaks to avoid unnecessary suffering and loss of life.^34^ Shah et al. shared their experience within an international peer research mentorship program, which was meant to equip medical students with the tools to become competent researchers and communicators upon graduation. The authors’ positive experiences within the program highlight the need for more widespread incorporation of such initiatives across medical schools globally.^35^ Equally important to obtaining research experiences are early clinical exposures to both specialties and geographic regions where medical students may envision themselves practicing post-graduation. In two articles we gain insight into rural medicine from a clerkship in rural Australia,^36^ and about mental health provision in rural settings through the interview of Dr. Van Gilder-Pierce.^37^ Finally, the Letter to the Editor acts as a reminder that “*Primum non nocere*” or “*do no harm*” should not only be applied to serving patients but also be a key commitment of healthcare providers to themselves—to prioritize mental and physical wellbeing, which will in turn aid in provision of better care to patients.^38^

The last decade of consistent and quality publications would not have been possible without the tireless efforts and hard work of all the members both past and present of the IJMS Editorial Team and Editorial Board. A special acknowledgement goes to the **Student Editor of the Year 2022:** Dr. Lourdes A. Medina Gaona, Instituto Tecnológico y de Estudios Superiores de Monterrey, Mexico; **Raising Star Student Editor 2022:** Dr. Rebecca N. Murerwa, University of Nairobi; Kenya; and the **Associate Editor of the Year 2022:** Dr. Sohaib Hasseb, James Cook University, Queensland, Australia. The IJMS looks forward to what the next decade will bring.
